# Great American Smokeout — November 21, 2019

**DOI:** 10.15585/mmwr.mm6845a1

**Published:** 2019-11-15

The American Cancer Society’s Great American Smokeout is an annual event that encourages smokers to make a plan to quit smoking (https://www.cancer.org/healthy/stay-away-from-tobacco/great-american-smokeout.html). The 44th annual Great American Smokeout will occur on November 21, 2019.

In the more than 50 years since the first Surgeon General’s report on the health consequences of smoking, cigarette smoking among U.S. adults has declined by approximately two thirds (*1*). A report in this issue of *MMWR* documented that in 2018, 13.7% of U.S. adults were current cigarette smokers, which is the lowest prevalence recorded since monitoring began in 1965 (*2*). However, the report also found that 34.2 million adults still smoke cigarettes and that marked disparities in tobacco use persist across population groups (*2*).

Smoking remains the leading preventable cause of disease, disability, and death in the United States (*1*); however, smokers can and do quit smoking, and today there are more former smokers than current smokers (*1*,*2*). Among current U.S. adult smokers, nearly 70% want to quit smoking, and approximately half made a quit attempt in the past year (*2*,*3*). Using counseling and medications increases the chances of quitting (*3*). Support for quitting smoking is available at 800-QUIT-NOW (800-784-8669). CDC’s Tips From Former Smokers campaign (https://www.cdc.gov/tips) and the National Cancer Institute’s smokefree.gov (https://smokefree.gov/) offer additional resources.
